# Orientation Control of Perfluorosulfonic Acid Films via Addition of 1,2,4-Triazole during Casting

**DOI:** 10.3390/polym16172533

**Published:** 2024-09-07

**Authors:** Tatsuya Miyajima, Susumu Saito, Takumi Okuyama, Satoshi Matsushita, Tetsuji Shimohira, Go Matsuba

**Affiliations:** 1 Innovative Technology Research Center, AGC Inc., 1-1 Suehirocho, Turumi-ku, Yokohama 230-0045, Japan; tatsuya.miyajima@agc.com (T.M.); susumu.saito@agc.com (S.S.); takumi.okuyama@agc.com (T.O.); satoshi.matsushita@agc.com (S.M.); tetsuji.shimohira@agc.com (T.S.); 2 Department of Organic Materials Science, Yamagata University, 4-3-16 Jonan, Yonezawa 992-8510, Japan

**Keywords:** perfluorosulfonic acid polymer, fuel cell, orientation, ion-cluster, X-ray analysis

## Abstract

Perfluorosulfonic acid (PFSA) polymers are used as electrolyte membranes in polymer electrolyte fuel cells. To investigate the effect on proton conductivity through structural orientation control, we added 1,2,4-triazole to PFSA films during casting to impart anisotropy to the ion-cluster structure of the films. The proton conductivities of the films were found to be high in the film-surface direction and low in the film-thickness direction. Structural analysis using small-angle X-ray scattering suggested that the anisotropy in proton conductivity was due to anisotropy in the ion-cluster structure, which in turn was attributed to the formation of a phase-separated structure via strong bonding between sulfonic acid groups and 1,2,4-triazole during cast film formation and the surface segregation of fluorine. We expect the findings of this study to aid in the fabrication of PFSA films with controlled ion clusters.

## 1. Introduction

Polymer electrolyte fuel cells (PEFCs) are environment-friendly and efficient power sources that operate at low temperatures. PEFCs are used in various applications, such as portable power sources, automobiles, and residential cogeneration systems [1]. Additionally, PEFCs have recently attracted attention in the field of heavy-duty vehicles and are expected to be used in sustainable energy systems [2]. PEFCs generate electricity by oxidizing hydrogen to hydrogen ions at the anode; electrons and protons pass through the circuit and electrolyte membrane, respectively, and the electrons and protons then react with oxygen at the cathode to generate water. The electrolyte membrane is highly proton-conductive, and its function is to separate hydrogen and oxygen. Perfluorosulfonic acid (PFSA) polymers are primarily used for this purpose because of their high proton conductivity and excellent physical and chemical durability [3,4,5]. Furthermore, PFSA polymers are also used as coating resins for catalysts in the cathode and anode of PEFCs owing to their high proton conductivity and oxygen permeability [5]. However, the proton conductivity of a PFSA polymer is significantly affected by its water content and decreases with decreasing humidity [5]. Meanwhile, at the same humidity, proton conductivity is significantly affected by the sulfonic acid group concentration or ion exchange capacity (IEC) of the PFSA polymer. This is because each sulfonic acid group contains approximately the same amount of water at a specific humidity [5,6]. Therefore, the most effective method to improve proton conductivity is to increase the IEC; however, this decreases the mechanical strength of the polymer [5].

Numerous studies have been conducted on the structures of monomers with sulfonic acid groups, including long-chain monomers such as Nafion^TM^ (Chemours, Wilmington, DE, USA) and FORBLUE^TM^ (AGC Inc., Tokyo, Japan), short side-chain monomers such as in the polymer Aquivion^®^ (Solvay S.A., Brussels, Belgium), and polymers fabricated under modified annealing conditions [5,7,8]. As oxygen permeability is required for catalyst layer applications, systems in which monomers with cyclic structures are copolymerized have been reported [9,10,11]. PFSA polymers have a hydrophilic sulfonic acid group and a hydrophobic fluorine matrix in their molecular chain; therefore, in a hydrated state, the hydrophilic ion cluster and the main-chain backbone of the hydrophobic fluorine matrix form a microphase-separated structure. The ion cluster plays a major role in proton conductivity, depending on the water content of the polymer. Therefore, the structure and behavior of these ion clusters have been extensively studied by many researchers [4,5,12,13,14,15,16,17,18,19,20,21,22,23]. The anisotropy of the phase-separated structure also affects its physical properties; therefore, the proton conductivity of uniaxially stretched films [21,22] and the changes in structure and proton conductivity that result from compression [23] have also been investigated from the perspective of physical deformation. However, in catalyst-coated resins, the anisotropy that can be imparted to ion clusters via physical deformation, such as stretching or compression, is limited because the films are formed by coating a dispersion of the catalyst and ionomer. The structures of ion clusters in thin-film PFSA resins have been extensively studied [5,24,25,26,27,28,29,30], and some examples of oriented ion-cluster structures have been reported; owing to the nanometer-scale thickness of catalyst-coated resins, the interaction between Pt and sulfonic acid groups strongly affects the ion-cluster structure. Thus, it is important to investigate the relationship between the proton conductivity and ion-cluster structure of the PFSA polymer and to control the structural orientation. Therefore, in addition to physical deformation, controlling the anisotropy of ion-cluster structures using casting methods is considered to have a wide range of applications.

Kim et al. have conducted studies on acid–base composite membranes, in some of which they investigated the effect of various additives on the properties of Nafion^TM^ to improve its performance as an electrolyte membrane [31,32,33,34,35,36,37,38]. It was inferred that a highly efficient proton conduction path was maintained in systems containing 1,2,4-triazole. With residual additive being typically undesirable, an investigation was also conducted after the removal of 1,2,4-triazole; notably, there was still an increase in conductivity compared to standard Nafion membranes [38]. The presence of a layered structure before the removal of the additive was suggested, implying an anisotropic structure. However, there was no discussion of proton conductivity or ion-cluster anisotropy. If the orientation of the ion-cluster structure can be controlled during cast film formation, it may lead to the future control of physical properties.

In this study, we used a PFSA polymer with a higher IEC than the polymer used by Kim et al. [38] and 1,2,4-triazole as an additive to cast an electrolyte membrane, followed by treatment to remove the additive. We then conducted structural analysis focused on the ion-cluster structure and investigated the mechanism affecting the formation of the ion-cluster structure.

## 2. Experimental

### 2.1. Materials

**Figure 1:**
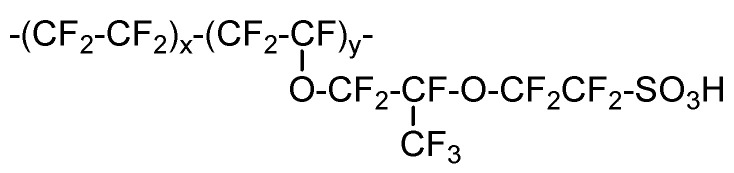
Molecular structure of a perfluorosulfonic acid (PFSA) polymer.

PFSA films of 25 µm or 50 µm thicknesses, fabricated by casting and a polymer solution of PFSA comprising a solvent composed of water/ethanol = 40/60 by weight and a PFSA concentration of 5 wt% (–SO_3_H-type), were supplied by AGC Inc. (Japan). The polymer structures of PFSA in the film and polymer solution were identical, with IEC values of 1.1 meq/g (Figure 1). Ethanol (purity > 99.0%) was purchased from Kanto Chemical Co., Inc. (Chuo-ku, Tokyo, Japan). *N*-methyl-2-pyrrolidone (NMP; purity: 99.0%), HCl (product code: 20010-0352), and KOH (product code: 39040-0301) were purchased from Junsei Chemical Co., Ltd. (Chuo-ku, Tokyo, Japan). 1,2,4-Triazole (purity > 99.0%) was purchased from Tokyo Chemical Industry Co., Ltd. (Chuo-ku, Tokyo, Japan). Water was sourced from a Milli-Q^®^ IQ 7005 purification system. A Pt/C catalyst (TEC10E50E) was purchased from Tanaka Kikinzoku Kogyo (Chuo-ku, Tokyo, Japan). The ionomer dispersion IC100 was supplied by AGC Inc. (Japan). Gas diffusion layers (GDLs; X0086 IX92 CX320 and X0086 T10X13) were purchased from Freudenberg-NOK (Weinheim, Germany; Tokyo, Japan). Vapor-grown carbon fibers (VGCF-Hs) were purchased from Showa Denko K. K. (Minato-ku, Tokyo, Japan).

### 2.2. Sample Preparation

The PFSA film was used as supplied (referred to as STD film). STD film is an EtOH/water-based cast film, and no triazole is used during film formation. The cast film was prepared as follows. First, 40 g of NMP was mixed with 40 g of the PFSA polymer solution. Then, 0.1 g of 1,2,4-triazole was added, and the mixture was held at 80 °C for 16 h; the amount of triazole used in the film formation corresponded to 0.65 equiv of the sulfonic acid groups in PFSA. The solution was cast onto a perfluoroalkoxy dish and subjected to heat treatment at 80 °C for 24 h, followed by 130 °C for a further 24 h, to obtain a triazole-treated cast film. The cast film was then washed with water at 80 °C for 2 h and subjected to H_2_O_2_ treatment at 80 °C for 16 h to remove the undesirable components NMP and 1,2,4-triazole. Then, it was washed with hydrochloric acid and water at 80 °C for 2 h, dried with filter paper three times, and air-dried to obtain the triazole-treated film. Triazole-treated films with thicknesses of 25 and 120 μm were prepared with the same composition ratio.

### 2.3. Characterization

The water vapor adsorption properties of the membranes were evaluated using a BELSORP 18-HT gas/vapor adsorption analyzer (MicrotracBEL Corp., York, PA, USA). Samples were first placed in glass tubes and vacuum-dried at 120 °C for 12 h to remove pre-adsorbed moisture. The water adsorption performance was then calculated from the changes in the mass of the samples equilibrated at 50 °C by introducing water vapor to achieve conditions of different relative humidities (RHs). The proton conductivity in the film-surface direction was calculated from AC impedance measurements using the four-terminal method. The LCR meter E4980AL (Keysight Technologies Inc., Santa Rosa, CA, USA) was used for the measurements. Measurements were performed using a 5 mm wide sample of a 25 μm thick cast film under the following conditions: frequency range of 20–300 kHz, voltage of 1 V, temperature of 80 °C, and RH of 50%. As it was difficult to directly evaluate the proton conductivity in the direction of the film thickness, we evaluated the power generation properties. Catalyst-coated membranes (CCMs) were fabricated using a 25 μm thick cast film to evaluate power generation properties. The CCMs were fabricated on both the anode and cathode sides using the decal transfer method. Anode and cathode catalyst inks were prepared by mixing a Pt/C catalyst (TEC10E50E), ethanol, and an ionomer dispersion (IC100) in an ultrasonic homogenizer. The ratio of the ionomer to the carbon support was 0.8. After coating and drying on ethylene–tetrafluoroethylene copolymer films at an amount of 0.4 mg-Pt/cm^2^ for both the anode and cathode, the catalyst layers were transferred to the electrolyte membrane by hot-pressing. The CCMs were used to fabricate the membrane electrode assembly (MEA). A GDL (X0086 IX92 CX320) with a hydrophobic microporous layer (MPL) was used as the anode side. A previous study by Tanuma reported the use of GDLs with hydrophilic MPLs as cathode-side GDLs [39]; therefore, a slurry of VGCF-Hs, ionomer dispersion (IC100), water, and ethanol was prepared in an ultrasonic homogenizer and coated onto a hydrophobic-treated GDL (X0086 T10X13) without an MPL at a solid content of 3.0 mg/cm^2^ to use as the cathode-side GDL. An MEA with an active area of 25 cm^2^ was assembled in the test cell by hot-pressing (150 °C, 2 MPa) the anode-side GDL in contact with the anode catalyst layer and the cathode-side GDL in contact with the cathode catalyst layer. Polarization curves (I–V curves) and internal resistance (IR) were measured at 80 °C and 100% RH at the anode and cathode inlets. The stoichiometric ratios for hydrogen and air were set to 1.4 and 2.0, respectively.

Small-angle X-ray scattering (SAXS) was performed on the cast films. The SAXS measurements were conducted at the BL05XU beamline in SPring-8 at JASRI (Sayo-gun, Hyogo, Japan) with an incident X-ray wavelength of 1 Å and a PILATUS 1M detector (DECTRIS A.G., Baden, Switzerland). The film-surface direction was measured using conventional transmission measurements. Microbeam X-rays were used to obtain information across the film-thickness direction; the microbeam was approximately 20 μm in width and 10 μm in height, and a stage for grazing-incidence measurements was used for the sample stage. The sample was cut to a width of 3 mm and placed on a glass slide. The horizontal alignment of the sample was adjusted before measuring the center of the sample in the film-thickness direction. Since the sample thickness was required for measurement in the film-thickness direction, a 50 μm thick STD film and 120 μm thick triazole-treated film were used. Additionally, prior to the H_2_O_2_ treatment, SAXS measurements across the film-surface direction were performed using a NANO-PIX (RIGAKU Corporation, Akishima, Japan) with an incident X-ray wavelength of 1.54 Å (Cu-Kα) and a HyPix-6000 detector (RIGAKU Corporation). The obtained data were subjected to circular averaging to obtain a one-dimensional profile. The scattering vector, *q*, was 4πsinθ/λ (2θ: scattering angle, λ: X-ray wavelength). The temperature and humidity in the beam hatch of SPring-8 at JASRI were stable, being within 27 ± 1 °C and 20 ± 3% relative humidity during measurements. The temperature and humidity during lab measurements were within 25 ± 1 °C and 30 ± 3% relative humidity during measurements.

The surface compositions of the films were analyzed using X-ray photoelectron spectroscopy (XPS). The measurements were performed using a PHI 5000 VersaProbe III spectrometer (ULVAC-PHI Inc., Chigasaki, Japan). A monochromatic Al-Kα beam (1486.6 eV) with a diameter of 100 μm was used, and the photoelectron extraction angle was set to 15° with respect to the sample. To suppress changes in the surface conditions under high vacuum, the sample was cooled to −140 °C with liquid nitrogen, and the elemental composition ratios of the sample surface were calculated.

## 3. Results and Discussion

### 3.1. Properties of PFSA Films

**Figure 2:**
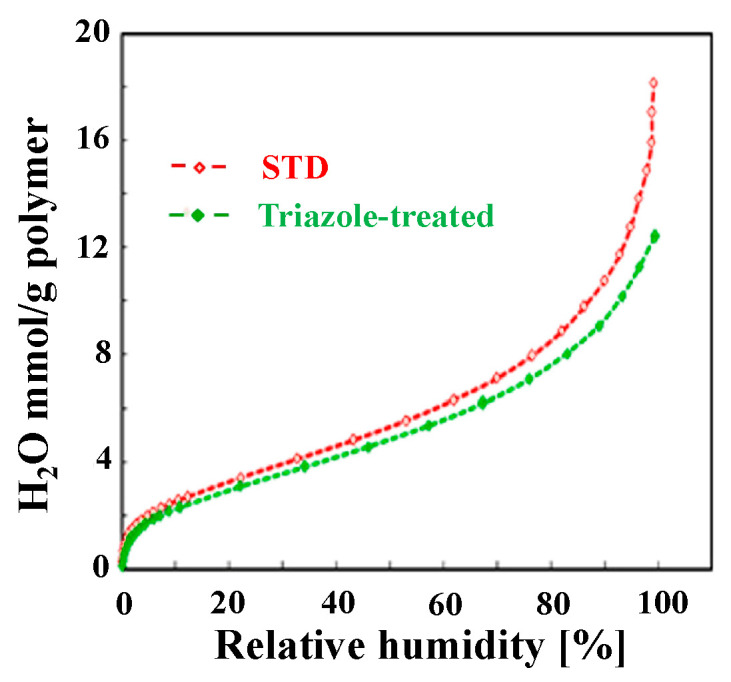
Relationship between membrane water uptake and relative humidity of cast films at 50 °C.

**Figure 3:**
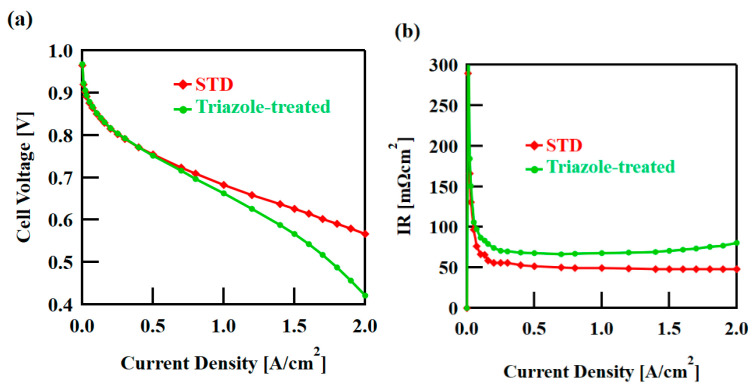
Fuel cell performance of membrane electrode assemblies at 100% RH and 80 °C. (**a**) Cell voltage and (**b**) internal resistance (IR).

Figure 2 shows the humidity dependence of the water vapor adsorption ability of the cast films. The triazole-treated film adsorbed slightly less water in the high-humidity range than the STD film; however, overall, there was no obvious difference. This was significantly influenced by the fact that the IECs were the same for both samples. Table 1 shows the proton conductivity in the film-surface direction at 80 °C and 50% RH, as determined by the four-terminal method. The triazole-treated film exhibited approximately 1.5 times higher proton conductivity than the STD film. The conductivity is expected to vary with humidity, but we suspect that anisotropy will be maintained. As the proton conductivity in the film-thickness direction was difficult to measure using the four-terminal method, the power-generation performance of the MEAs was compared at 80 °C and 100% RH (Figure 3). The triazole-treated film exhibited a large voltage drop at high current densities and a high overall resistance. At a typical current density of 1.2 A/cm^2^, the resistance of the triazole-treated film was approximately 1.4 times that of the STD film, indicating that the proton conductivity was low in the film-thickness direction. We also attempted measurements at 30% RH, but the resistance of the triazole-treated film was high and appropriate voltage measurements could not be performed.

Kim et al. reported an increase in proton conductivity in both the surface and thickness directions of the film [38]; however, in this experiment, the proton conductivity increased in the film-surface direction and decreased in the film-thickness direction, and a clear anisotropy was observed. Compared to Kim et al., this system uses PFSA with higher IEC, less triazole, and the addition of NMP. The addition of NMP was intended to increase the swelling of both the main and side chains and to reduce the amount of triazole used to make the interaction between the sulfonic acid groups and the triazole more effective. It is possible that the difference in the anisotropy of proton conductivity was due to a significant change in the interaction between the sulfonic acid group and the triazole. The difference in the drying process of the films may also be a factor, given that λ was higher in the system with triazole in the Kim et al. experiment.

The dynamic viscoelastic behavior of the cast films showed differences in the relaxation temperature of *T*_α_ due to ion clusters (Figure S1 and Table S1 in the supporting information). This also suggested that there was a difference in the ion cluster structure between the STD film and the triazole-treated film.

Although the triazole-treated and STD films adsorbed the same amount of water in the bulk state, the triazole-treated film exhibited higher conductivity in the film-surface direction and lower conductivity in the film-thickness direction; these properties are undesirable, as high proton conductivity in the film-thickness direction is important for PEFC performance. It was also found that the casting method using triazole could impart anisotropic proton conductivity across the film thickness and surface. To investigate the reason behind the difference in proton conductivity, the SAXS profiles were analyzed for the ion-cluster structure in the film-thickness and film-surface directions.

### 3.2. X-ray Structural Analysis

**Figure 4:**
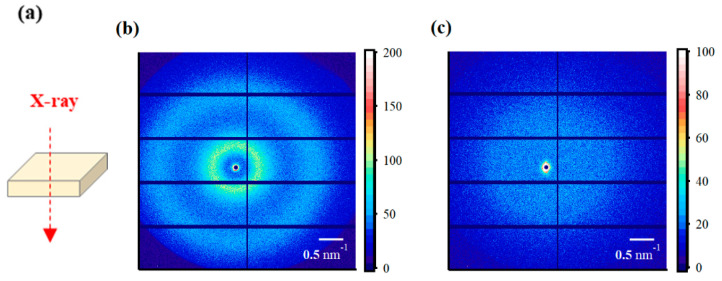
(**a**) Schematic diagram of X-ray incidence direction for small-angle X-ray scattering (SAXS) analysis from the film-surface direction. SAXS images from the film-surface direction of the (**b**) STD film and (**c**) triazole-treated film.

**Figure 5:**
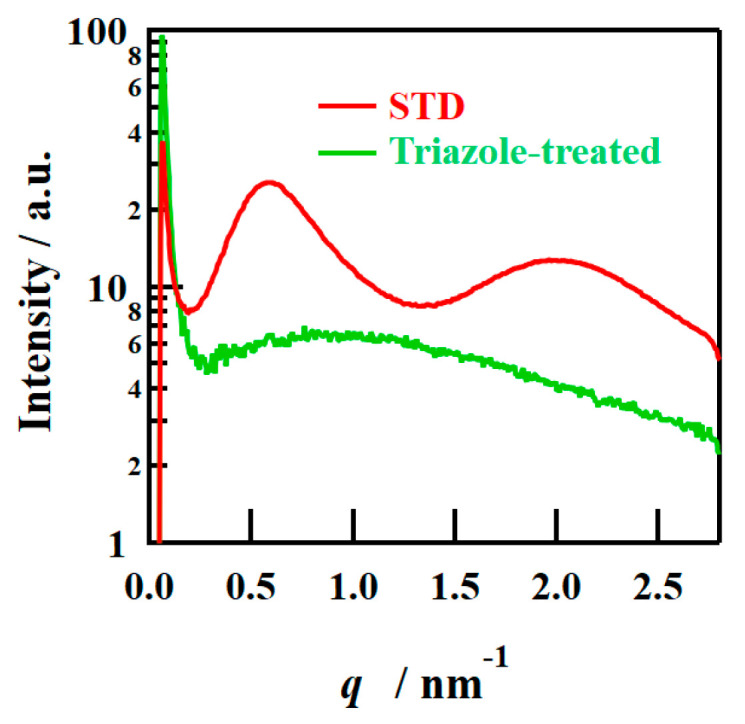
One-dimensional (1D) SAXS profiles from the film-surface direction of PFSA films.

The SAXS images obtained using incident X-rays on the film-surface direction of the PFSA films are shown in Figure 4. While two types of isotropic scattering were detected for the STD film, only unclear scattering was observed for the triazole-treated film. Figure 5 shows the one-dimensional SAXS profiles obtained by azimuthal averaging of the SAXS images. The one-dimensional SAXS profiles were corrected for transmission, air scattering, and film thickness, and the scattering intensities were compared. The profile of the STD film showed a matrix-derived peak (*q* = ~0.6 nm^–1^) and an ion-cluster-derived peak (*q* = ~2.0 nm^−1^) [5]. However, only weak and broad peak were detected for the triazole-treated film. Table 2 lists the *D* values calculated from the peak position *q*_peak_ using the formula *D* = 2π/*q*_peak_.

*D* values indicate the distance between crystals or ion clusters [5]. The triazole-treated film exhibited larger inter-cluster distances than the STD film, although this is an approximate value from very broad peak.

**Figure 6:**
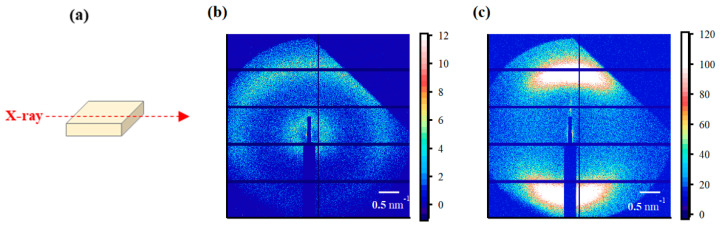
(**a**) Schematic diagram of X-ray incidence direction for SAXS analysis from the film-thickness direction. SAXS images from the film-thickness direction of the (**b**) STD film and (**c**) triazole-treated film.

**Figure 7:**
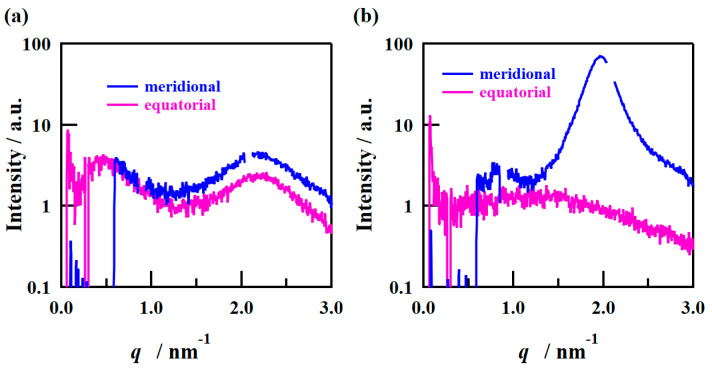
1D SAXS profiles from the film-thickness direction of the (**a**) STD film and (**b**) triazole-treated film.

**Figure 8:**
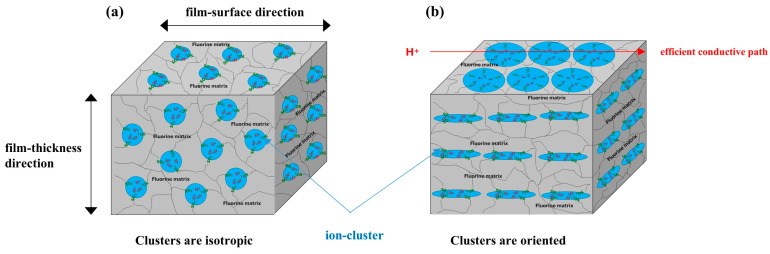
Schematic image of ion-cluster structure. (**a**) STD film and (**b**) triazole-treated film.

The SAXS images obtained using incident X-rays from the film-thickness direction of the PFSA films are shown in Figure 6. The STD film was nearly isotropic, with two types of scattering detected: matrix-derived and ion-cluster-derived. The ion-cluster-derived scattering was slightly weaker at the bottom of the image, possibly because some of the scattering was absorbed by the glass slides used to fix the specimens. In contrast, for the triazole-treated film, a spot peak was detected in the meridian direction, and no clear scattering was detected in the equatorial direction. The spot-like scattering was similar to that reported for uniaxial stretching [19,22,40,41]. Although direct comparisons are difficult, Park et al. discuss uniaxial stretching and water transport and mention the effect of such anisotropy on SAXS images and proton conductivity [22]. Figure 7 shows the SAXS profiles obtained by converting them to one-dimensional directions in the meridional and equatorial directions. The meridional and equatorial directions were integrated over a ±5° range for the right side and top of the SAXS image, respectively. The discontinuous part where no scattering is obtained in the meridian direction corresponds to the beam stopper or detector dead area. For the STD film, the peak intensity derived from ion clusters was slightly lower in the equatorial direction; however, the peak positions were the same. In contrast, for the triazole-treated film, strong ion-cluster-derived peaks were detected in the meridional direction, no matrix-derived peaks were observed, and very weak broad scattering was observed in the equatorial direction. A comparison of the equatorial scattering patterns of the STD film and the triazole-treated film shows a similar trend to that of the film-surface direction. The *D* values calculated from the peak positions are listed in Table 3. The distance between ion clusters in the equatorial direction of the triazole-treated film is an approximate value from very broad peak; however, the distance between ion clusters is larger in the triazole-treated film than in the STD film, both in the equatorial and meridional directions. From the above results, it was inferred that the STD film exhibited isotropic scattering in both the film-surface and film-thickness directions, but the triazole-treated film exhibited scattering that was strongly oriented in the film-thickness direction. Figure 8 shows a schematic of the cluster structure assumed based on the SAXS profile analysis. As the scattering of the STD film was isotropic in both the film-surface and film-thickness directions, it was inferred that the clusters were also isotropic. However, considering that the triazole-treated film had an unclear scattering profile in the film-surface direction and a clear spot scattering profile in the film-thickness direction, we assumed that long elliptical or flat clusters existed in a stacked state in the film-surface direction. Therefore, it was inferred that efficient conductive paths formed in the film-surface direction of the triazole-treated film; however, the proton conductivity was low in the film-thickness direction because of the thick fluorine matrix layer with low conductivity. 

Cast films were also prepared with either no triazole or 10 times the amount of triazole using the same processing method, and the proton conductivity in the film-surface direction was measured. The proton conductivity of the film without triazole was comparable to that of the STD film, whereas the conductivity of the film with 10 times the triazole was comparable to that of the triazole-treated film (Table S2 in the supporting information). Microbeam SAXS analysis of these samples showed that the SAXS pattern trends were the same with and without triazole, indicating that the addition of triazole had a significant effect on the orientation of the ion clusters (Figure S2 in the supporting information). It would be desirable to verify the assumed structure in real space using techniques such as TEM or AFM; however, it is difficult to obtain data that can be discussed by ensuring appropriate pretreatment and measurement environment because this polymer needs to be analyzed in a water-containing state.

### 3.3. Mechanism Estimation

**Figure 9:**
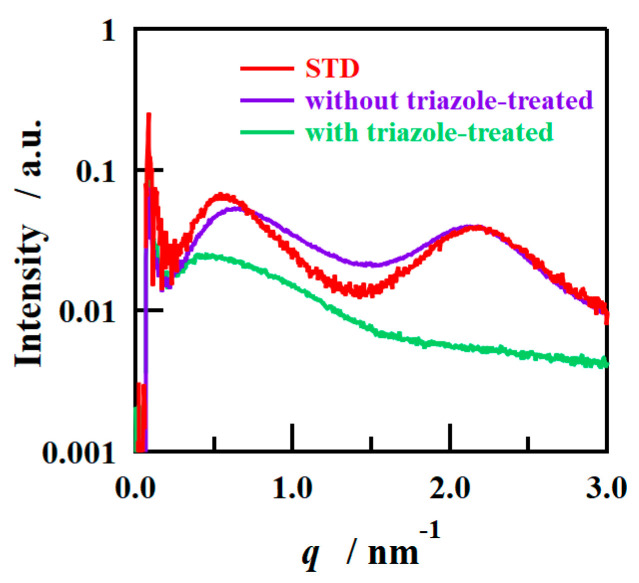
SAXS profiles of STD film and PFSA films before H_2_O_2_ treatment.

To investigate the mechanism behind determining the orientation of the ion-cluster structure in the triazole-treated film, SAXS measurements were conducted on the cast films prior to H_2_O_2_ treatment. Figure 9 shows the SAXS profiles in the film-surface direction of the cast films prepared with and without the addition of 1,2,4-triazole and without H_2_O_2_ treatment, and Table 4 shows the *D* values calculated from the peak positions. Figure 9 also shows the SAXS profile of the STD film measured under the same conditions for comparison. The SAXS profiles of the STD film and the film without triazole were similar, and the film with triazole added had weak scattering intensity, which also showed a similar tendency to the triazole-treated film after H_2_O_2_ treatment. In other words, it was inferred that the structure with oriented ion clusters (Figure 8) was formed after cast film formation and that the oriented structure was retained after H_2_O_2_ treatment. The peak position of the triazole-treated film before H_2_O_2_ treatment is close to the peak position of the crystal-derived peak of the STD film, but the peak is assumed to be derived from the cluster because the crystal-derived peak was not detected after H_2_O_2_ treatment, and the cluster is large because it contains NMP and triazole. Care must be taken to ensure accurate comparisons, though, due to insufficient control of the measurement environment. In the triazole-added film, the distance between clusters is larger than that after H_2_O_2_ treatment, possibly because the clusters contain NMP as well as water and triazole. The NMP and triazole are removed by H_2_O_2_ treatment, and the ionic clusters are reconstituted with water and sulfonic acid groups, so it is inferred that the inter-cluster distance becomes smaller after H_2_O_2_ treatment. Triazole molecules were expected to be present in the ionic clusters and form strong bonds with the sulfonic acid groups, promoting the development of phase-separated structures during film formation [32,38]. In addition, the low surface free energy of fluorine led to the segregation of a fluorine-rich matrix layer on the surface. This resulted in the formation of a layered structure consisting of fluorine- and ion-cluster-rich layers, which cannot be formed using conventional casting methods. We also assumed that the phase separation between the ion clusters and the fluorine matrix was caused by triazole; consequently, the matrix-derived peaks that are usually observed in normal membranes were not confirmed by SAXS for the triazole-treated film. Although the order of phase separation is slightly different, the formation of layered structures in fluoropolymers has been reported in thin films of block copolymers [42]. To verify this hypothesis, we conducted XPS measurements on the outermost surface of the sample and calculated the S/F ratio by focusing on the differences between the sulfonic acid groups/fluorine matrixes. Table 5 shows the S/F ratios of the STD and triazole-treated films before H_2_O_2_ treatment. As the S/F value calculated from the composition ratio of the STD film was 0.1, both films exhibited lower S/F values than that expected from the bulk, and it was inferred that a fluorine-rich layer was formed on the outermost layer. No difference in S/F ratio was observed between samples, possibly because the segregation of fluorine to minimize the surface free energy at the air interface was stronger than the effect of additives such as triazole. The segregation of fluorine in the outermost layer was the same among the samples. It was inferred that the strength of the acid–base interaction between the triazole and sulfonic acid groups greatly contributed to the formation of the layered structure. Depth XPS analysis was also attempted to verify the difference between with and without triazole; however, it was difficult to properly evaluate the composition owing to the damage caused by X-ray analysis and sputtering.

## 4. Conclusions

To impart anisotropy to the physical properties of PFSA, we cast PFSA films with 1,2,4-triazole as an additive, resulting in films with high proton conductivity in the film-surface direction and low proton conductivity in the film-thickness direction. From X-ray structural analysis, it was inferred that this was due to the anisotropy of the ion-cluster structure. The formation of this anisotropic ion-cluster structure was inferred to result from the strong bonding of sulfonic acid groups and triazole molecules during cast film formation, the development of a phase-separated structure, and the surface segregation of fluorine. Although this technology is not suitable for direct application as PEFC electrolyte membranes, it can potentially be used for the development of PFSA membranes with controlled ion cluster structures. For example, a film formed between hydrophilic/fluorophilic nano-patterned substrates can be oriented in the film-thickness direction; therefore, it will be useful as a highly proton-conductive electrolyte membrane. Furthermore, because ion clusters are oriented, anisotropy can be imparted to swelling when water is added, even in the bulk film.

## Figures and Tables

**Table 1:** Proton conductivity of cast films at 80 °C and 50% relative humidity (RH), as determined by the four-terminal method.

	Proton Conductivity (S/cm)
STD film	0.055
Triazole-treated film	0.083

**Table 2:** *D* values for PFSA films in the film-surface direction.

	*D* for Matrix (nm)	*D* for Ion Cluster (nm)
STD film	10.6	3.14
Triazole-treated cast film	–	6.8 *

* Approximate value from very broad peak in Figure 5.

**Table 3:** *D* values for PFSA films in the film-thickness direction.

	*D* for Matrix in Equatorial Direction (nm)	*D* for Matrix in Meridional Direction (nm)	*D* for Ion Cluster in Equatorial Direction (nm)	*D* for Ion Cluster in Meridional Direction (nm)
STD film	13.0	–	2.87	2.89
Triazole-treated cast film	–	–	5 *	3.19

* Approximate value from very broad peak in Figure 7b.

**Table 4:** *D* values for STD film and the PFSA films before H_2_O_2_ treatment.

	*D* for Matrix (nm)	*D* for Ion Clusters (nm)
STD film	11.1	2.90
Cast film treated without triazole before H_2_O_2_ treatment	9.7	2.96
Cast film treated with triazole before H_2_O_2_ treatment	–	12.6

**Table 5:** S/F ratios on the surfaces of STD film and the PFSA films before H_2_O_2_ treatment, as determined by XPS.

	S/F (Atom)
STD film	0.028
Cast film treated without triazole before H_2_O_2_ treatment	0.024
Cast film treated with triazole before H_2_O_2_ treatment	0.024

## Data Availability

Raw data were generated at AGC Inc. Derived data supporting the findings of this study are available from the corresponding author G.M. on request.

## Supplementary Materials

The following supporting information can be downloaded at https://www.mdpi.com/article/10.3390/polym16172533/s1, Figure S1: Relationship between storage modulus, loss modulus, tanδ and temperature of PFSA cast films; Figure S2: SAXS images from the film-thickness direction of PFSA films. (a) Schematic diagram of X-ray incidence direction, (b) cast film treated without triazole, (c) Cast film treated with 10 times the amount of triazole; Figure S3: 1D SAXS profiles from the film-thickness direction. (a) Cast film treated without triazole, (b) cast film treated with 10 times the amount of triazole; Table S1: Transition temperature of PFSA cast films; Table S2: The proton conductivity at 80 °C and 50% relative humidity of PFSA films; Table S3: *D* values for PFSA films in the film-thickness direction of the cast films without triazole treatment and with 10 times the amount of triazole treatment.
