# Supplementary material for: Orientation Control of Perfluorosulfonic Acid Films via Addition of 1,2,4-Triazole during Casting
Source: Polymers (Basel). 2024 Sep 7;16(17):2533. doi: 10.3390/polym16172533 (PMC11397893; doi:10.3390/polym16172533)
Supplement: Supplementary file 1 [file polymers-16-02533-s001.zip › polymers-3191484-supplementary.pdf]

(Supporting Information)

Orientation Control of Perfluorosulfonic Acid Films via Addition of 1,2,4-Triazole during Casting

Tatsuya Miyajima [1,2], Susumu Saito [1], Takumi Okuyama [1], Satoshi Matsushita [1], Tetsuji Shimohira [1] and Go Matsuba [2,*]

1 Innovative Technology Research Center, AGC Inc., 1-1 Suehirocho, Turumi-ku, Yokohama 230-0045, Japan
2 Department of Organic Materials Science, Yamagata University, 4-3-16 Jonan, Yonezawa 992-8510, Japan
* Correspondence: gmatsuba@yz.yamagata-u.ac.jp; Tel.: +81-238-26-3053

1. Thermal mechanical transition of PFSA cast filme

Thermal mechanical transitions of PFSA cast films were collected by DMA (DVA200, IT-Keisoku K.K.). The measurement conditions were a temperature range of -100°C to 250°C at 2°C/min, a constant frequency of 1 Hz. The DMA result is shown in Figure S1. In both samples, β transition originating from the main chain motion is observed around -60°C to -20°C, and α transition originating from ion clusters is observed around 70°C to 130°C [5]. T_α calculated from the peak position of tanδ are shown in Table S1. T_α is higher in the triazole-treated film compared to the STD film, suggesting that the state of ion clusters is different in these membranes.

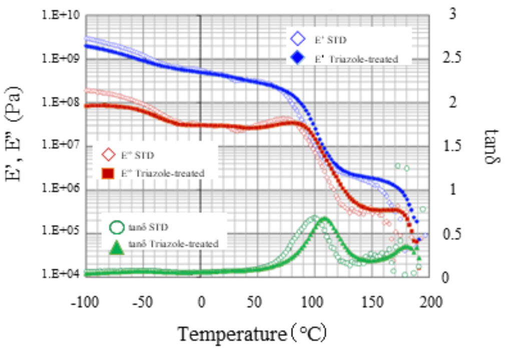

Figure S1. Relationship between Storage modulus, Loss modulus, tanδ and temperature of PFSA cast films.

Table S1. Transition temperature of PFSA cast films.

Sample	T_α (°C)
STD	99
Triazole-treated	108

2. Proton conductivity and SAXS analysis of PFSA membranes with different amount of 1,2,4-toriazole.

Cast films were also prepared with either no triazole or 10 times the amount of triazole using the same processing method, and the proton conductivity in the film-surface direction was measured. Table S2 shows the conductivity in the film-surface direction. The proton conductivity of the film without triazole was comparable to that of the STD film, whereas the conductivity of the film with 10 times the triazole was comparable to that of the triazole-treated film.

Structural analysis of these films by microbeam SAXS in the film-thickness direction was performed. Figure S2 shows the SAXS images. The SAXS image trends were the same with and without triazole, indicating that the addition of triazole had a significant effect on the orientation of the ion-cluster. Figure S3 shows the SAXS profiles obtained by converting them to one-dimensional directions in the meridional and equatorial directions. The *D* values calculated from the peak positions are listed in Table S3. The distance between ion clusters in the meridional direction of the triazole-treated film is an approximate value from very broad peak, but the distance between ion clusters is larger in the samples using triazole, both in the equatorial and meridional directions. In terms of anisotropy, it is more strongly expressed in the scattering pattern than in the *D* value.

Table S2. The proton conductivity at 80°C and 50% relative humidity of PFSA films

	Proton conductivity (S/cm)
Cast film treated without triazole	0.055
Cast film treated with 10-times the amount of triazole	0.083

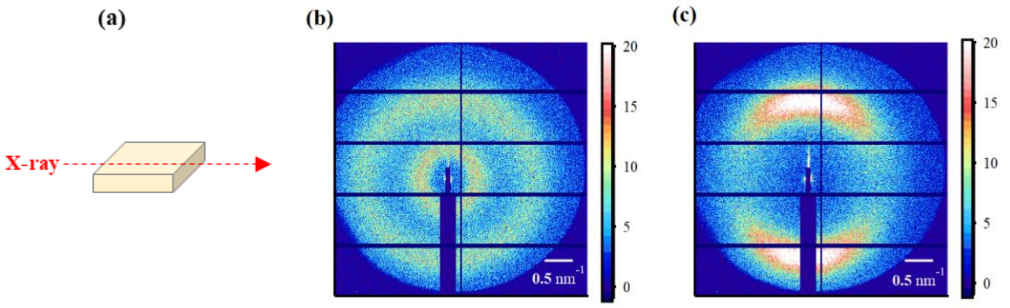

Figure S2. SAXS images from film-thickness direction of PFSA films (a) Schematic diagram of X-ray incidence direction, (b)Cast film treated without triazole (c) Cast film treated with 10-times amount of triazole.

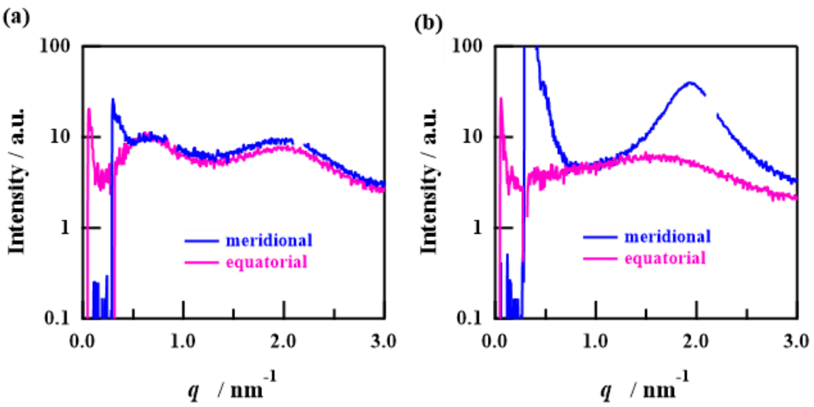

Figure S3. 1D SAXS profiles from the film-thickness direction. (a)Cast film treated without triazole (b) Cast film treated with 10-times amount of triazole.

Table S3. *D* values for PFSA films in the film-thickness direction of the cast films without triazole treatment and with 10-times amount of triazole treatment.

	D for ion cluster in equatorial direction (nm)	*D* for ion cluster in meridional direction (nm)
Cast film treated without triazole	3.17	3.17
Cast film treated with 10-times the amount of triazole	4*	3.24

*Approximate value from very broad peak in Fig. S3
